# Bone metastases and immunotherapy in patients with advanced non-small-cell lung cancer

**DOI:** 10.1186/s40425-019-0793-8

**Published:** 2019-11-21

**Authors:** Lorenza Landi, Federica D’Incà, Alain Gelibter, Rita Chiari, Francesco Grossi, Angelo Delmonte, Antonio Passaro, Diego Signorelli, Francesco Gelsomino, Domenico Galetta, Diana Giannarelli, Hector Soto Parra, Gabriele Minuti, Marcello Tiseo, Maria Rita Migliorino, Francesco Cognetti, Luca Toschi, Paolo Bidoli, Francovito Piantedosi, Luana Calabro’, Federico Cappuzzo

**Affiliations:** 1 Department of Oncology and Hematology, AUSL Romagna, Ravenna, Italy; 2 Fondazione Ricerca Traslazionale, Rome, Italy; 3 Oncologia Medica B, Policlinico Umberto I, Rome, Italy; 4 Medical Oncology, Santa Maria della Misericordia Hospital, Perugia, Italy; 5 Division of Medical Oncology, IRCCS Ca’ Granda Ospedale Maggiore Policlinico, Milan, Italy; 6 Istituto Scientifico Romagnolo per lo Studio e la Cura dei Tumori, Meldola, Italy; 7 Division of Thoracic Oncology, IEO, European Institute of Oncology IRCCS, Milan, Italy; 8 Fondazione IRCCS Istituto Nazionale dei Tumori, Milan, Italy; 9 Oncologia Medica, Policlinico S. Orsola – Malpighi, Bologna, Italy; 10 Oncologia Medica Toracica, IRCCS Istituto Tumori “Giovanni Paolo II”, Bari, Italy; 11 IRCCS - Regina Elena National Cancer Institute, Rome, Italy; 12 AOU Policlinico Vittorio Emanuele, Catania, Italy; 13 UO Oncologia Medica, Azienda Usl Toscana Nord Ovest, Livorno, Italy; 14 Medical Oncology Unit, University Hospital, Parma, Italy; 15 UOSD Pneumologia Oncologica, Azienda Ospedaliera San Camillo Forlanini, Rome, Italy; 16 Humanitas Cancer Center, Rozzano, Milan, Italy; 17 Oncology Unit, ASST, Ospedale S. Gerardo, Monza, Italy; 18 U.O.S.D. DH Pneumoncologico, A.O. Dei Colli Monaldi - Cotugno-CTO, Naples, Italy; 19 Medical Oncology and Immunotherapy, Center for Immuno-Oncology, University Hospital of Siena, Siena, Italy

**Keywords:** Bone metastases, Nivolumab, Immunotherapy, PD-L1, Non-small-cell lung cancer

## Abstract

**Background:**

Bone metastases (BoM) are a negative prognostic factor in non-small-cell lung cancer (NSCLC). Beyond its supportive role, bone is a hematopoietic organ actively regulating immune system. We hypothesized that BoM may influence sensitivity to immunotherapy.

**Methods:**

Pretreated non-squamous (cohort A) and squamous (cohort B) NSCLCs included in the Italian Expanded Access Program were evaluated for nivolumab efficacy according to BoM.

**Results:**

Cohort A accounted for 1588 patients with non-squamous NSCLC, including 626 (39%) with (BoM+) and 962 (61%) without BoM (BoM-). Cohort B accounted for 371 patients with squamous histology including 120 BoM+ (32%) and 251 (68%) BoM- cases. BoM+ had lower overall response rate (ORR; Cohort A: 12% versus 23%, *p* <  0.0001; Cohort B: 13% versus 22%, *p* = 0.04), shorter progression free survival (PFS; Cohort A: 3.0 versus 4.0 months, p <  0.0001; Cohort B: 2.7 versus 5.2 months, p <  0.0001) and overall survival (OS; Cohort A: 7.4 versus 15.3 months, p <  0.0001; Cohort B: 5.0 versus 10.9 months, *p* < 0.0001). Moreover, BoM negatively affected outcome irrespective of performance status (PS; OS in both cohorts: p < 0.0001) and liver metastases (OS cohort A: p < 0.0001; OS Cohort B: *p* = 0.48). At multivariate analysis, BoM independently associated with higher risk of death (cohort A: HR 1.50; cohort B: HR 1.78).

**Conclusions:**

BoM impairs immunotherapy efficacy. Accurate bone staging should be included in clinical trials with immunotherapy.

## Introduction

In the last few years, improvements in cancer biology and immune system knowledge significantly prolonged survival of patients with metastatic non-small cell lung cancer (NSCLC) [1–3]. Agents targeting the programmed death-1 receptor (PD-1)/ PD-ligand 1 (PD-L1) pathway, also named immune checkpoint inhibitors (ICIs), have emerged as powerful therapeutic strategy in different settings [1–7]. Nivolumab, pembrolizumab and atezolizumab are three recommended options for patients who progress after platinum-doublet chemotherapy, whereas pembrolizumab is the standard front line for untreated patients with PD-L1 expression > 50% [1–8]. As a consequence, the proportion of patients still candidate for exclusive chemotherapy is gradually decreasing. At present, PD-L1 expression is the only validated biomarker adopted in clinical practice for selecting NSCLC candidate for immunotherapy [8]. Several other biomarkers are under investigation with Tumor Mutational Burden (TMB) as the one closest to a routine adoption [9]. Recently, some clinical factors such as performance status and metastatic sites, emerged as potential predictors for immunotherapy efficacy [10–12]. In a retrospective study conducted in 201 Asian patients and treated with nivolumab, poor performance status (ECOG PS > 2) and presence of lung or liver metastases independently associated with shorter PFS [11]. Another study evaluated organ specific response rate to second or subsequent line nivolumab in 52 patients with higher response observed in lymph nodes (28%) followed by primary lung lesions (16%). Interestingly, among patients with bone metastases, 75% had progressive bone lesions at the time of tumor progression [12]. This data suggest that efficacy of immunotherapy can be modulated by tumor microenvironment, which differs among organs.

Because of its large surface area and highly vascular supply, bone is a common site of metastatic spread in NSCLC [13, 14]. Implementation of diagnostic tools coupled with survival improvement have resulted in a raised incidence of bone metastases, with 20–30% of NSCLC patients presenting with bone lesions at diagnosis and an additional 35–40% of cases developing bone metastases during the course of their disease [15]. Patients with bone disease frequently experience skeletal-related events entailing severe pain and deterioration in their performance status (PS) and quality of life. Not surprisingly, bone metastases negatively affect overall survival [13]. In addition to its supportive role, bone is a hematopoietic organ. Several evidences showed that bone marrow plays active functions in regulating immune system and trafficking of immune cells, including regulatory T cells, conventional T cells, B cells, dendritic cells, natural killer T cells, neutrophils, myeloid-derived suppressor cells and mesenchymal stem cells [16]. Therefore, bone marrow is an immune regulatory organ potentially influencing response to immunotherapy.

However, none of the randomized studies specifically investigated the consequences of bone involvement in patients exposed to checkpoint inhibitors or stratified patients according to presence of bone metastases. Aim of the present study was to assess whether efficacy of nivolumab was influenced by presence of bone disease in a large pretreated, metastatic, NSCLC.

## Material and methods

### Study design, patients and treatment

The trial was conducted in two cohorts (Cohort A = non-squamous-cell histology, *N* = 1588; cohort B = squamous-cell histology, *N* = 371) of advanced NSCLC patients treated in 153 Italian centers and included in the nivolumab Expanded Access Program (EAP) from April 2015 to September 2016. The Italian EAP was a prospective, single-arm, open-label trial intended to provide early access to nivolumab. Details of main inclusion and exclusion criteria and treatment have been previously published [17, 18]. Briefly patients aged ≥18 years, with histologically or cytologically diagnosis of non-squamous or squamous NSCLC, pretreated with at least one systemic therapy for advanced disease and an Eastern Cooperative Oncology Group (ECOG) PS < 2, were included onto the study if they had no evidence of carcinomatous meningitis or serum positivity for HIV, HBV or HCV and no prior therapy checkpoint inhibitor. In both cohorts nivolumab 3 mg/kg was administered intravenously every 2 weeks for ≤24 months. Patients included in the analysis had received at least 1 dose of nivolumab. This study was conducted in accordance with the Declaration of Helsinki (1964) and International Conference of Harmonization Guidelines for Clinical Practice and was approved by the appropriate Institutional Review Board/Independent Ethics Committee. All patients provided written informed consent before treatment.

### Outcome measures

Tumor response was assessed using Response Evaluation in Solid Tumors (RECIST) criteria version 1.0 [19]. Investigator-assessed objective response rate (ORR), progression free survival (PFS) and overall survival (OS) were evaluated. Patients were monitored for adverse events (AEs) using the National Cancer Institute Common Terminology Criteria for Adverse Events v4.0.

### Statistical analysis

Efficacy and safety analyses were conducted in all patients who received at least one nivolumab dose. ORR, PFS, OS and safety were evaluated. Chi-square measured association between patient characteristics and ORR. PFS was calculated as the time from the start of nivolumab treatment until evidence of progressive disease or death whichever occurred first. PFS and OS were estimated using Kaplan-Meier method and 95% confidence intervals (CIs) were derived using Hosmer and Lemeshow approach. Differences between survival curves were evaluated with log rank test. A Cox regression model was used to explore the association between patient characteristics and survival times; Hazard Ratios (HRs) with 95% CIs were reported. When performing a multivariate analysis, a stepwise procedure was used based on Wald statistics, enter and remove values set to 0.05 and 0.10, respectively.

## Results

### Patient populations

#### Cohort A

Characteristics of non-squamous cohort are illustrated in Table 1. Among the 1588 patients, 626 (39%) had bone metastases (BoM+ group) and 962 (61%) had no evidence of bone involvement (BoM- group). BoM+ patients were younger than BoM- (*p* = 0.001) with a significantly lower percentage of individuals older than 75 years (*p* < 0.0001). ECOG PS was 0 in 34% of BoM+ and 45% in BoM- (p < 0.0001). Brain and liver metastases were significantly more frequent in the BoM+ group (p = 0.001 and *p* < 0.00001 for brain and liver, respectively). No difference in terms of gender, smoking status and number of prior systemic therapies was observed in BoM+ and BoM-.

**Table 1:** Characteristics in cohorts A and B according to bone involvement

Characteristic	Cohort A			Cohort B		
	Non-squamous BoM+ (N/%)	Non-squamous BoM- (N/%)	p	Squamous BoM+ (N/%)	Squamous BoM- (N/%)	p
Total patients	626 (39)	962 (61)		120 (32)	251 (68)	
Median age (year/range)	65 (29–89)	67 (27–87)	0.001	67 (31–83)	68 (31–91)	0.06
Patients ≥75 years	67 (11)	165 (17)	< 0.0001	18 (15)	52 (21)	0.19
Gender						
• Male	401 (64)	628 (65)		96 (80)	202 (81)	0.91
• Female	225 (36)	334 (35)	0.62	24 (20)	49 (19)	
ECOG PS						
• 0	213 (34)	435 (45)	< 0.0001	36 (30)	98 (39)	0.25
• 1	354 (56)	461 (48)		77 (64)	138 (55)	
• 2	57 (9)	51 (5)		7 (6)	15 (6)	
• Unk	2 (1)	15 (2)		–	–	
Metastatic site						
• liver	178 (28)	149 (16)	< 0.00001	25 (21)	38 (15)	0.14
• brain	191 (30)	218 (23)	0.001	16 (13)	21 (8)	0.17
Previous therapies						
• 1	256 (41)	359 (37)	0.07	54 (44)	108 (43)	0.67
• 2	176 (28)	281 (29)		37 (31)	83 (33)	
• 3	118 (19)	162 (17)		20 (17)	48 (19)	
• > 3	74 (12)	154 (16)		9 (8)	12 (5)	
Smoking status						
• Never smoker	134 (21)	171 (18)	0.06	13 (11)	18 (7)	0.14
• Former smoker	308 (49)	457 (48)		69 (57)	156 (62)	
• Active smoker	134 (21)	226 (23)		23 (19)	60 (24)	
• Unk	50 (8)	108 (11)		15 (13)	17 (7)	
*EGFR* status						
• Mutated	47 (8)	55 (6)	0.10			
• Wild-type	514 (82)	779 (81)				
• Unk	65 (10)	128 (13)				
*KRAS* status						
• Mutated	91 (15)	115 (12)	0.23			
• Wild-type	132 (21)	192 (20)				
• Unk	403 (64)	655 (68)				
*BRAF* status						
• Mutated	5 (1)	6 (1)	0.54			
• Wild-type	85 (14)	114 (12)				
• Unk	536 (85)	842 (87)				
*ALK* status						
• Mutated	5 (1)	11 (1)	0.51			
• Wild-type	407 (65)	645 (67)				
• Unk	214 (34)	306 (32)				
*ROS1* status						
• Mutated	3 (1)	1 (1)	0.29			
• Wild-type	142 (23)	207 (21)				
• Unk	481 (76)	754 (78)				

*Unk* Unknown

Percentage of *EGFR*, *KRAS*, *BRAF*, *ALK* and *ROS1* mutations was similar in the two groups. Among BOM+ patients, 264 (42.1%) had received palliative radiotherapy to the bone.

#### Cohort B

Among the 371 patients with squamous histology, 120 (32%) were BoM+ and 251 BoM- (68%). Age, gender, PS, and presence of liver or brain metastases did not significantly differ between BoM+ and BoM- patients (Table 1). Thirty-eight (31.6%) BoM+ had received palliative radiotherapy to the bone.

### Efficacy in cohort A

**Fig. 1:**
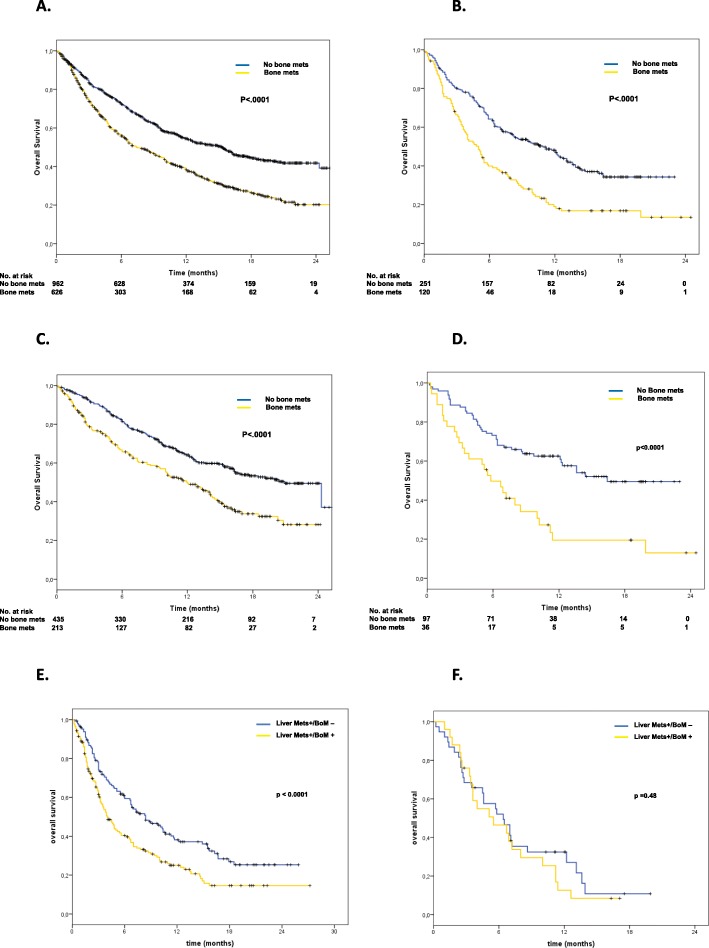
OS in the two cohorts, in patients with PS = 0 and in patients with liver metastases. **a**: In all non-squamous patients, OS was 7.4 versus 15.3 months in BoM+ and BoM- (< 0.0001), respectively. **b**: In all squamous patients, OS was 5.0 versus 10.9 months in BoM+ and BoM- (< 0.0001), respectively. **c**: In non-squamous patients with PS = 0, OS was 12.0 versus 20.9 months (p < 0.0001) in patients BoM+ and BoM-, respectively. **d**: In squamous patients with PS = 0, OS was 5.8 versus 16.4 months (p < 0.0001) in patients BoM+ and BoM-, respectively. **e**: In non-squamous patients with liver metastases, OS was 4.0 versus 8.4 months (*p* < 0.0001) in patients BoM+ and BoM-, respectively. **f**: In squamous patients with liver metastases, OS was 5.5 versus 6.4 months (p = 0.48) in patients BoM+ and BoM-, respectively

As illustrated in Table 2, outcome of patients with bone metastases was particularly poor. BoM+ patients had significantly lower ORR (12% versus 23%, *p* < 0.0001), shorter PFS (3.0 versus 4.0 months, p < 0.0001) and OS (7.4 versus 15.3 months, p < 0.0001) than BoM-. Figures 1a and Additional file 1A show the difference in PFS and in OS between BoM+ and BoM- patients. At 12 months, only 15% of BoM+ patients did not progress versus 27% in the BoM- group (*p* < 0.0001). OS rate at 12 months was also in favor of BoM- patients (38% in BoM+ versus 55% in BoM-, p < 0.0001)*.*

**Table 2:** Efficacy in cohorts A and B and according to bone involvement

Parameter	All non Squamous (N/%)	All Squamous (N/%)	Non SquamousBoM+ (N/%)	Non-SquamousBoM- (N/%)	p	Squamous BoM+ (N/%)	Squamous BoM- (N/%)	p
Response								
• CR	12/1	4/1	7/1	5/1	< 0.0001*	0/0	4/2	0.04*
• PR	278/17	65/18	66/11	212/22		15/13	50/20	
• SD	414/26	108/28	137/22	277/29		25/21	83/33	
• PD	818/52	189/50	393/63	425/44		78/65	111/44	
• NV	66/4	5/3	23/3	43/4		2/2	3/1	
Median PFS (months/range)	3.0 (2.9–3.1)	4.2 (3.4–5.0)	3 (2.9–3.1)	4 (3.5–4.5)	< 0.0001	2.7 (2.2–3.2)	5.2 (4.3–6.0)	< 0.0001
12 months PFS (%)	23	27	15	27	< 0.0001	15	31	< 0.0001
Median OS (months/range)	11.3 (10.2–12.4)	7.9 (6.2–9.6)	7.4 (6.0–8.8)	15.3 (13.2—17.4)	< 0.0001	5 (3.9–6.1)	10.9 (8.4–13.4)	< 0.0001
12 months OS (%)	48	39	38	55	< 0.0001	19	48	< 0.0001

*p value was calculated in CR + PR versus SD + PD

In order to assess whether PS, liver or brain metastases could drive the poor outcome of BoM+ individuals, we analyzed ORR, PFS and OS in these specific subgroups. As illustrated in Additional file 2, presence of BoM statistically associated with poor outcome in terms of ORR, PFS and OS irrespective of the three variables considered (Fig. 1 c,e; Additional file 1 C-E and Additional file 3 A-B). We further restricted our analysis to the 615 patients receiving nivolumab in second-line setting only. Also in this subgroup, ORR, PFS and OS were significantly worse in BoM+ patients (Additional file 4 A,B; Additional file 2). Finally, we analyzed the outcome of the 102 patients harboring *EGFR* mutations according to presence of BoM (Additional file 5 A,B; Additional file 2), with similar results.

### Efficacy in cohort B

As illustrated in Table 2, outcome of BoM+ patients was similar to what observed in the non-squamous cohort. BoM+ patients had significantly lower ORR (13% versus 22%, *p* = 0.04), shorter PFS (2.7 versus 5.2 months, *p* < 0.0001; Additional file 1B) and OS (5.0 versus 10.9 months, p < 0.0001; Fig. 1b). At 12-months PFS was 15% in BoM+ and 31% in BoM- (*p* = 0.001) while 12-months OS was 19% in BoM+ versus 48% in BoM- (p < 0.0001)*.*

Efficacy analyses according to PS or presence or liver or bone metastases confirmed that the worse outcome observed in individuals with skeletal involvement was not related to a lower PS of the BoM+ patients or to concomitant spread into liver or brain (Fig.1 b,d,f; Additional file 1 D,F; Additional file 3 C,D). Analogous results were observed in patients treated with nivolumab in second-line setting (Additional file 6 and Additional file 7).

To better define the role of bone metastases in a different population of pretreated NSCLC, we re-analyzed data from patients enrolled in the phase II METROS trial [20]. In this group of oncogene addicted population, presence of bone metastases negatively affected both PFS and OS (p 0.02 and 0.04, respectively. Data not shown).

### Univariate and multivariate analyses

Clinical variables potentially influencing survival were included in a univariate model (Table 3). The variables resulted significant, were further included in a multivariate model. In both cohorts, among factors included in the univariate model, PS, liver metastases and bone metastases independently associated with higher risk of death in the multivariate model (HRs in BoM+: 1.50 in non-squamous and 1.78 in squamous, p < 0.0001 for both cohorts). The same results were obtained when considering these factors for PFS and ORR (Additional file 8 and Additional file 9).

**Table 3:** Univariate and multivariate analyses for OS in cohorts A and B combined

	Cohort A				Cohort B			
Factors	Univariate analysis HR (95% C.I.)	*p*	Multivariate analysis HR (95% C.I.)	*p*	Univariate analysis HR (95% C.I.)	*p*	Multivariate analysis HR (95% C.I.)	*p*
Age (≥ 65 vs ≤ 65)	0.9 (0.80–1.04)	0.18	–	–	1.13 (0.86–1.47)	0.38	–	–
Gender								
(male vs female)	1.03 (0.89–1.18)	0.73	–	–	1.20 (0.87–1.67)	0.27	–	–
ECOG PS								
1 vs 0	1.59 (1.37–1.83)	< 0.0001	1.45 (1.24–1.69)	< 0.0001	1.59 (1.20–2.11)	0.001	1.51 (1.14–2.00)	0.04
2 vs 0	3.47 (2.71–4.45)	< 0.0001	3.09 (2.38–4.02)	< 0.0001	2.53 (1.54–4.17)	< 0.0001	2.46 (1.49–4.05)	< 0.0001
Smoking habits								
Current/former vs never	0.85 (0.72–1.00)	0.05	–	–	1.27 (0.79–2.07)	0.32	–	–
Brain mets (yes vs no)	1.24 (1.06–1.43)	0.006	–	–	1.07 (0.70–1.63)	0.75	–	–
Liver mets (yes vs no)	1.84 (1.58–2.15)	< 0.0001	1.64 (1.39–1.93)	< 0.0001	1.58 (1.16–2.15)	0.004	1.43 (1.01–1.95)	0.03
Bone mets (yes vs no)	1.67 (1.46–1.91)	< 0.0001	1.50 (1.30–1.73)	< 0.0001	1.90 (1.47–2.47)	< 0.0001	1.78 (1.37–2.31)	< 0.0001
Previous CT Lines								
2 vs 1	0.87 (0.73–1.03)	0.10	–	–	0.91 (0.61–1.22)	0.54	–	–
> 2 vs 1	1.03 (0.88–1.21	0.67			0.73 (0.53–1.02)	0.06		

### Additional analyses

In order to define the impact of palliative radiotherapy to the bone, we analyzed data considering all BoM+ patients (non-squamous and squamous) as divided into two groups: patients with bone metastases treated with RT (BoM+/RT+, *N* = 302) and patients with bone metastases and no prior RT (BoM +/RT-, *N* = 444). No differences in terms of OS, PFS and ORR were observed (Additional file 10). Further, we evaluated early deaths (intended as death within the first 3 months of treatment) and early progressions (intended as progression within the first 3 months of treatment) in the whole study population (non-squamous plus squamous, *N* = 1959) according to bone metastases (BoM+, *N* = 746; BoM- = 1213) and to prior RT. Both early deaths and early progressions were significantly higher in BoM + patients included in the EAP nivolumab program and were not influenced by prior RT (Additional file 11). Finally, we performed the same analysis considering the METROS cohort. In such study, early progressions events resulted numerically higher in patients with bone metastases (Additional file 11).

### Safety

Summary of AEs occurring in > 1% of patients is reported in Additional file 12. In cohort A any grade or grade 3–4 AEs were respectively 31 and 7% in BoM+ and 34 and 7% in BoM-. Differences were not statistically significant. The most common grade 3/4 treatment-related AEs were fatigue/asthenia (2%), anemia (1%), increased transaminases (2%), increased lipase/amylase (1%), dyspnea (1%), and pneumonitis (1%) in BoM+ patients, and fatigue/asthenia (2%), pain (1%), and dyspnea (1%) in BoM- patients. Discontinuation rate was 88% (*n* = 553) in BoM+ and 78% (*n* = 747) in BoM-. Treatment-related (TR) AEs leading to discontinuation occurred in 24 (4%) patients with bone metastasis and 41 (5%) patients without bone metastasis. Similar results were observed in the cohort B, where grade 3–4 gastrointestinal AEs occurred in 3% of BoM+ and < 1% in BoM-. BoM+ had endocrine grade 3–4 AEs in 5% versus < 1% in BoM-. TRAEs leading to discontinuation were reported in 16 (2.1%) of BoM+ and 63 (5.2%) in BoM-. Selected TRAEs were managed using protocol-defined toxicity management algorithms. No treatment-related deaths occurred.

## Discussion

While ICIs have shown significant efficacy in controlling visceral metastases in several malignancies, their specific efficacy in patients with bone metastases is not well understood [10–12]. To the best of our knowledge, this is the largest study investigating whether presence of bone metastases influences immunotherapy efficacy in NSCLC. BoM+ patients had poor outcome for any efficacy end-point, irrespective of tumor histology, patient PS, concomitant spread into the liver or brain, or prior palliative radiotherapy in the bone, showing that organ-specific metastases are relevant factors in individual candidate to immunotherapy.

Distant metastases, particularly in the liver or in the brain, negatively affect survival in NSCLC [21–23]. Even if clinical trials with immunotherapy generally included only patients with asymptomatic and pretreated brain metastases, immunotherapy seems effective in controlling intracranial disease [24, 25]. In addition, recent findings suggested that immunotherapy could be particularly effective in patients with hepatic localizations. In the IMPOWER 150 trial, a phase III study investigating efficacy of atezolizumab, a monoclonal antibody against PD-L1, in addition to carboplatin-paclitaxel-bevacizumab or to carboplatin-paclitaxel versus carboplatin-paclitaxel-bevacizumab combination, a remarkable OS improvement was observed in patients with liver metastases, raising the question whether site of disease is a relevant factor for immunotherapy [3]. The increasing interest in defining immunotherapy efficacy according to the site of metastatic [10–12], led us to focus our interest on the bone for two main reasons. The first one was the evidence that bone has a relevant role in modulating immune-response [16, 26]. Bone marrow contains high levels of multiple immune cells with relevant functions. It is now clear that bone marrow can supplant the secondary lymphoid tissue either as a site of primary immune response or memory response [16]. Thus, bone marrow is an immune regulatory organ, affecting systemic immunity and therapeutic efficacy of conventional treatments and immunotherapy [13]. The second reason relies on the evidence that presence of bone metastases is a negative prognostic factor in lung cancer. Literature data clearly indicated that skeletal involvement is associated with shorter survival [14]. Recently, a large phase III study confirmed that bone involvement is a negative prognostic factor. In the CheckMate 227 study, patients with bone metastases assigned to platinum-based chemotherapy had a median OS of only 8 months, shorter than in individuals without bone disease [27]. Nevertheless, none of the randomized trials with immunotherapy, including the CheckMate 227, stratified patients for site of metastases precluding any firm conclusion. In our study, two different cohorts of patients, accounting for a total of 1959 patients, received nivolumab in second or further line of therapy. In both cohorts, patients with bone metastases had significantly lower systemic response rate and significantly shorter PFS and OS. By analyzing the data, we first hypothesized that the negative outcome of BoM+ patients was related to the lower PS generally associated with bone metastases, or to coexistence of liver or brain metastases. Nevertheless, a detrimental effect was observed independently of PS or intracranial or hepatic involvement, thus suggesting a different mechanism than a simple PS deterioration or high tumor burden. Even if the lack of a control arm precluded the possibility to discriminate between predictive and prognostic role of bone metastases, data from the Checkmate 057 study, a phase III trial comparing nivolumab to docetaxel as second-line therapy in NSCLC, support the hypothesis that bone involvement could predict lower sensitivity to immunotherapy [28]. In this trial, among 161 patients with skeletal metastases, 86 received nivolumab and 75 docetaxel. Survival analysis showed that 26 out of 86 patients in the nivolumab arm versus 11 out of 75 in the docetaxel arm died within 3 months, and this difference was statistically significant (*p* = 0.019). Similarly, in our study, BoM+ patients had an excess in early progression and death reinforcing the hypothesis that immunotherapy cannot reverse the negative prognostic value of bone dissemination. In addition, a recent study in breast cancer mouse model showed that antitumor efficacy of PD-1 blockade is enhanced by concomitant administration of zoledronic acid, a biphosphonate-drug typically used in the treatment and prevention of pathologic fractures [26, 29]. All together these data support the concept of bone as an organ modulating sensitivity to immunotherapy. In our study, data on concomitant use of biphosphonates were not collected precluding us the possibility to explore whether such agents could also influence sensitivity to immunotherapy.

Other limitations of our study included its retrospective nature without a predefined method for bone assessment, the lack of information on bone involvement (single versus multiple lesions), the absence of a control arm without immunotherapy and the lack of information on PD-L1 expression and TMB status. PD-L1 expression was not required for study entry and lack of tumor tissue from patients included onto the study did not allow additional biomarker analyses. Indeed, further studies are warranted to define whether levels of PD-L1 expression or TMB differ in patients with or without bone metastases and whether the worse outcome of BoM+ patients depends on the status of the two biomarkers. Moreover, since all patients included in the present analyses were pretreated, it is not possible to define whether the same effect is present in first-line setting. Even with these limitations, the outcome of our patients was similar to what has been observed in clinical trials [5, 6].

Finally, whether anti-angiogenic agents could increase immunotherapy efficacy in BoM+ patients is a crucial question to address. In bone marrow, immature myeloid cells differentiate in myeloid-derived suppressor cells (MDSCs) and acquire immunosuppressive activity [16]. Among anti-cancer drugs potentially affecting the MDSC component, bevacizumab seems one of the most promising. In a recent study, Wallin et al. showed that combination of atezolizumab and bevacizumab increases intra-tumoral CD8 + T cells, suggesting that dual anti-VEGF and anti-PD-L1 inhibition improves antigen-specific T-cell migration [30]. Even if the IMPOWER 150 trial supported the synergistic effect of atezolizumab and bevacizumab combination, the efficacy of this strategy in BoM+ patients remains undefined and additional investigations are warranted [3].

## Conclusions

In conclusion, our data suggest that presence of BoM could impair immunotherapy efficacy. Additional studies should investigate biological mechanisms responsible for such effect, including whether PD-L1 expression or TMB could discriminate subpopulation of BoM+ patients benefiting from the treatment. Accurate bone staging should be included in clinical trials with immunotherapy.

## Supplementary information

**Additional file 1.** PFS in Cohort A and B according to bone metastases (A,B) and considering prognostic variables (C-F).

**Additional file 2.** Efficacy according to clinical characteristics in cohorts A.

**Additional file 3.** PFS and OS in Cohort A and B according to bone and brain metastases.

**Additional file 4.** PFS and OS in patients treated in second-line in Cohort A.

**Additional file 5 **PFS and OS in patients harboring *EGFR* mutations in Cohort A.

**Additional file 6.** PFS and OS in patients treated with nivolumab in second-line in cohort B.

**Additional file 7.** Efficacy according to clinical characteristics in cohorts B.

**Additional file 8.** Univariate and multivariate analyses for PFS in cohorts A and B combined.

**Additional file 9.** Univariate and multivariate analyses for ORR in cohorts A and B combined.

**Additional file 10.** Outcome to nivolumab in BOM+ patients according to prior RT.

**Additional file 11.** Early death and early progression in the whole study population according to bone metastases (BoM) and to prior palliative radiotherapy (RT) and in the METROS cohort.

**Additional file 12.** Treatment related adverse events in cohorts A and B.

## Data Availability

All the data analysed supporting the results reported in the article may be found/are archived at the Biostatistics Unit, Scientific Direction, IRCSS Regina Elena National Cancer Institute, Rome.

## Abbreviations

AE: Adverse event
ALK: Anaplastic lymphoma kinase
BoM: Bone metastases
BRAF: Serine/threonine-protein kinase B-Raf
CI: Confidence interval
CR: Complete response
EAP: Expanded Access Program
ECOG: Eastern Cooperative Oncology Group
EGFR: Epidermal growth factor receptor
HBV: Hepatitis B virus
HCV: Hepatitis B virus
HIV: Human immunodeficiency virus
HR: Hazard ratio
ICI: Immune checkpoint inhibitor
KRAS: Kirsten rat sarcoma viral oncogene homolog
MDSC: Myeloid-derived suppressor cell
NSCLC: Non-small-cell lung cancer
NV: Not valuable
ORR: Objective response rate
OS: Overall survival
PD: Progressive disease
PD-1: Programmed death-1 receptor
PD-L1: PD-ligand 1
PFS: Progression free survival
PR: Partial response
PS: Performance status
RECIST: Response Evaluation in Solid Tumors
ROS1: c-ros oncogene 1
SD: Stable disease
TMB: Tumor Mutational Burden
TRAE: Treatment related adverse event
Unk: Unknown
VEGF: Vascular endothelial growth factor
