# Supplementary material for: Bone metastases and immunotherapy in patients with advanced non-small-cell lung cancer
Source: J Immunother Cancer. 2019 Nov 21;7:316. doi: 10.1186/s40425-019-0793-8 (PMC6868703; doi:10.1186/s40425-019-0793-8)
Supplement: Supplementary file 1 — Additional file 1. PFS in Cohort A and B according to bone metastases (A,B) and considering prognostic variables (C-F). [file 40425_2019_793_MOESM1_ESM.doc]

**A. B.**


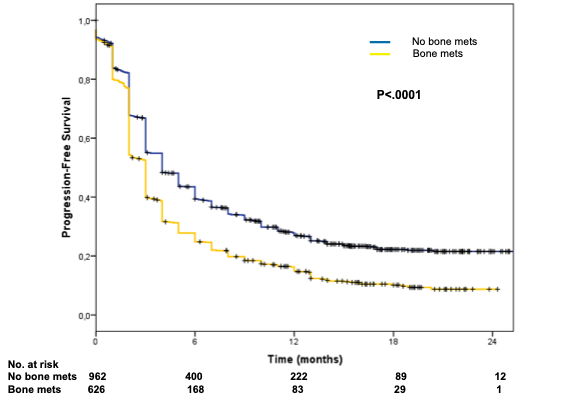

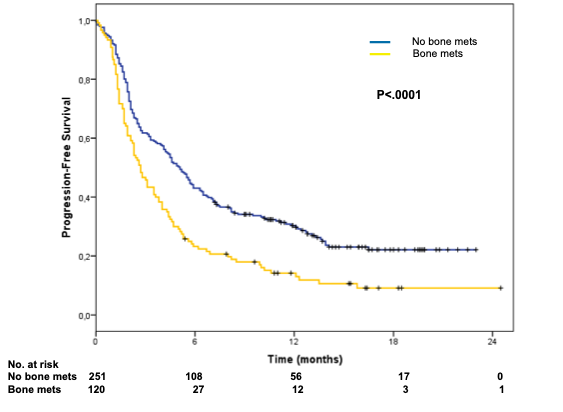


**C. D.**


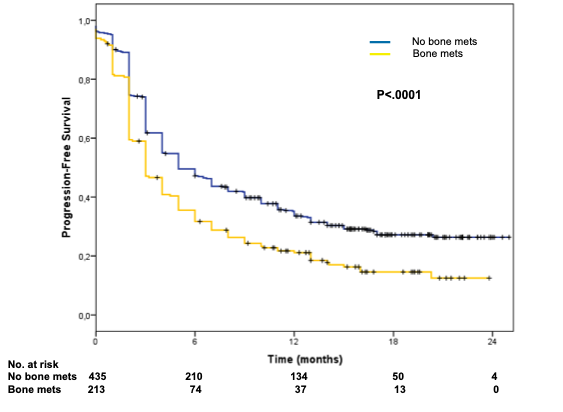

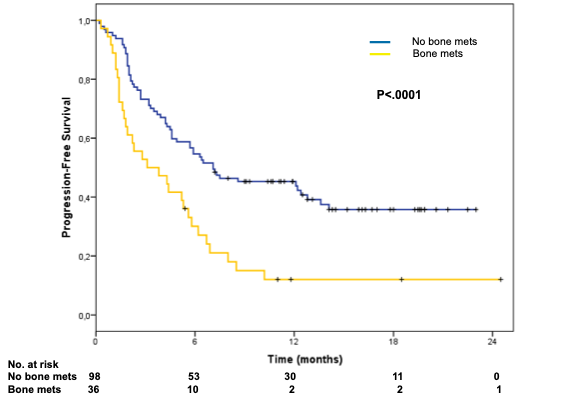


**E. F.**


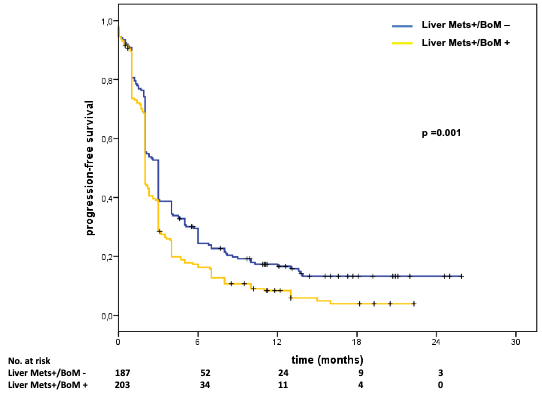

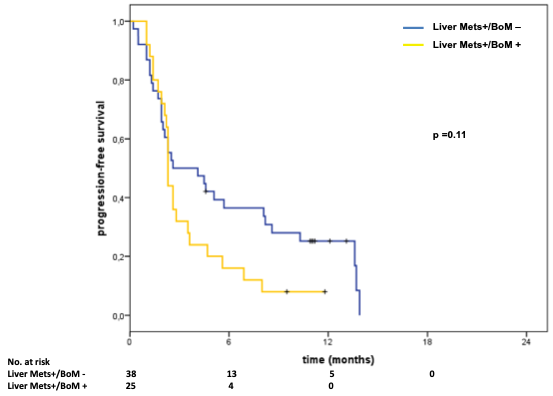


**Additional file 1: PFS in Cohort A and B according to bone metastases (A,B) and considering prognostic variables (C-F). A:** PFS in all non-squamous BoM+ was 3.0 versus 4.0 months in BoM- (p<0.0001). **B:** PFS in all squamous BoM+ was 2.7 versus 5.2 months in BoM- (p<0.0001). **C:** In non- squamous cohort and PS=0, PFS was 3.0 versus 5.0 months (p<0.0001) in patients BoM+ and BoM-, respectively. **D:** In squamous cohort and PS=0, PFS was 3.8 versus 7.2 months (p<0.0001) in patients BoM+ and BoM-, respectively. Figure S1E: In non-squamous cohort and liver metastases, PFS was 3.0 versus 4.0 months (p=0.001) in patients BoM+ and BoM-, respectively. **F:** In squamous cohort and liver metastases, PFS was 2.3 versus 2.6 months (p=0.11) in patients BoM+ and BoM-, respectively.
