# Supplementary material for: Bone metastases and immunotherapy in patients with advanced non-small-cell lung cancer
Source: J Immunother Cancer. 2019 Nov 21;7:316. doi: 10.1186/s40425-019-0793-8 (PMC6868703; doi:10.1186/s40425-019-0793-8)
Supplement: Supplementary file 2 — Additional file 2. Efficacy according to clinical characteristics in cohorts A. [file 40425_2019_793_MOESM2_ESM.doc]

**Additional file 2: Efficacy according to clinical characteristics in cohorts A**

| **Category** | **N/Total (%)** | **ORR*** | ***p*** | **mPFS, months** | ***p*** | **mOS, months** | ***p*** |
| --- | --- | --- | --- | --- | --- | --- | --- |
| **ECOG PS 0** |  |  |  |  |  |  |  |
| - Non Squamous BoM+ | 213/626 (34) | 13.6% | 0.0001 | 3.0 (2.3 – 3.7) | < 0.0001 | 12.2 (9.3 – 14.7) | < 0.0001 |
| - Non Squamous BoM- | 435/962(45) | 26.9 % |  | 5.0 (3.9 -6.0) |  | 20.9 (18.3 – 23.5) |  |
| **Liver Metastases** |  |  |  |  |  |  |  |
| - Non Squamous BoM+ | 178/626 (28) | 5.1% | 0.0003 | 2.0 (1.09 -2.05) | 0.008 | 4.0 (3.1 – 4.9) | < 0.0001 |
| - Non Squamous BoM- | 149/962 (16) | 17.5% |  | 3.0 (2.8 – 3.2) |  | 8.4 (5.9 – 10.9) |  |
| **Brain Metastases** |  |  |  |  |  |  |  |
| - Non Squamous BoM+ | 191/626 (30) | 9.4% | 0.0003 | 4.0 (3.3 – 4.7) | 0.001 | 12.8 (8.6 – 17.0) | < 0.0001 |
| - Non Squamous BoM- | 218/962 (23) | 22.9% |  | 3.0 (2.8 – 3.2) |  | 5.7 (4.4 – 7.0) |  |
| **Second line** |  |  |  |  |  |  |  |
| - Non Squamous BoM+ | 256/626 (41) | 15.0% | 0.03 | 3.0 (2.6-3.4) | <0.0001 | 7.3 (5.5 – 9.1) | < 0.0001 |
| - Non Squamous BoM- | 359/962 (37) | 22.0 % |  | 4.0 (3.1 - 4.9) |  | 15.0 (12.4 – 17.6) |  |
| **EGFR mutated** |  |  |  |  |  |  |  |
| - Non Squamous BoM+ | 47/626 (8) | 2.1% | 0.03 | 2.0 (1.5-2.5) | 0.14 | 5.4 (3.0-7.8) | 0.04 |
| - Non Squamous BoM- | 55/962 (6) | 14.5% |  | 3.0 (2.4 – 3.6) |  | 12.8 (0.2 – 25.4) |  |

*p value was calculated in CR+PR versus SD+PD
