# Supplementary material for: Bone metastases and immunotherapy in patients with advanced non-small-cell lung cancer
Source: J Immunother Cancer. 2019 Nov 21;7:316. doi: 10.1186/s40425-019-0793-8 (PMC6868703; doi:10.1186/s40425-019-0793-8)
Supplement: Supplementary file 3 — Additional file 3. PFS and OS in Cohort A and B according to bone and brain metastases. [file 40425_2019_793_MOESM3_ESM.doc]

**A. B.**


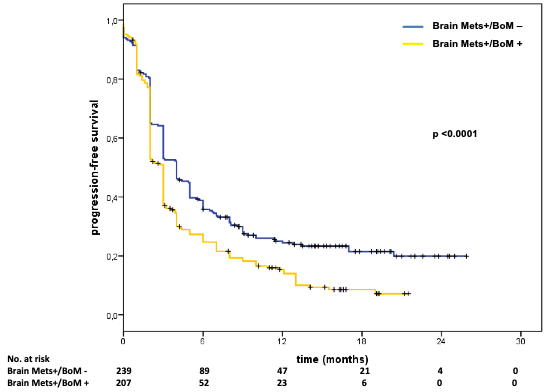

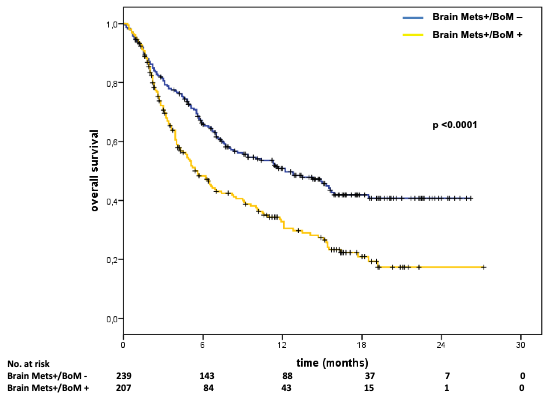


**C. D.**


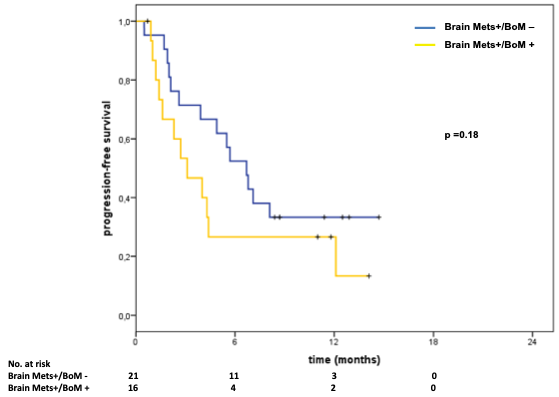

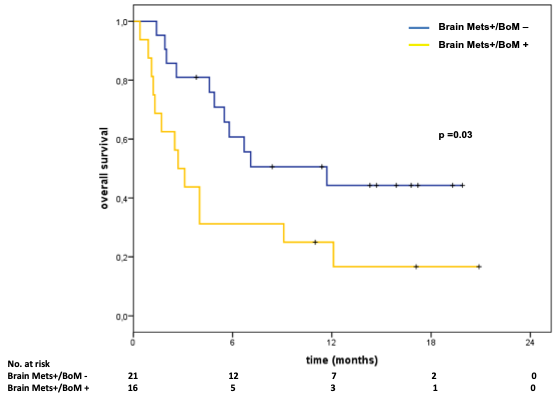


**Additional file 3: PFS and OS in Cohort A and B according to bone and brain metastases. A:** In non-squamous cohort and brain metastases, PFS was 3.0 versus 4.0 months (p=0.001) in patients BoM+ and BoM-, respectively. **B:** In non-squamous cohort and brain metastases, OS was 5.7 versus 12.8 months (p<0.0001) in patients BoM+ and BoM-, respectively. **C:** In squamous cohort and brain metastases, PFS was 2.5 versus 6.7 months (p=0.18) in patients BoM+ and BoM-, respectively. **D:** In squamous cohort and brain metastases, OS was 2.7 versus 11.7 months (p=0.03) in patients BoM+ and BoM-, respectively.
