# Supplementary material for: Bone metastases and immunotherapy in patients with advanced non-small-cell lung cancer
Source: J Immunother Cancer. 2019 Nov 21;7:316. doi: 10.1186/s40425-019-0793-8 (PMC6868703; doi:10.1186/s40425-019-0793-8)
Supplement: Supplementary file 4 — Additional file 4. PFS and OS in patients treated in second-line in Cohort A. [file 40425_2019_793_MOESM4_ESM.doc]

**A.**

**B.**

**Additional file 4: PFS and OS in patients treated in second-line in Cohort A**. **A:** PFS in patients treated with nivolumab in second-line was significantly shorter among BoM+ (3.0 months) than among BoM- (4.0 months, p<0.0001). **B:** OS in patients treated with nivolumab in second-line was significantly shorter among BoM+ (7.3 months) than among BoM- (15.0 months, p<0.0001).
