# Supplementary material for: Bone metastases and immunotherapy in patients with advanced non-small-cell lung cancer
Source: J Immunother Cancer. 2019 Nov 21;7:316. doi: 10.1186/s40425-019-0793-8 (PMC6868703; doi:10.1186/s40425-019-0793-8)
Supplement: Supplementary file 5 — Additional file 5 PFS and OS in patients harboring EGFR mutations in Cohort A. [file 40425_2019_793_MOESM5_ESM.doc]

**A.**

**B.**

**Additional file 5: PFS and OS in patients harboring *EGFR* mutations in Cohort A.** **A:** Presence of BoM resulted in shorter PFS in patients harboring *EGFR* mutations. **B:** In patients with *EGFR* mutations, OS was significantly shorter in BoM+ than in BoM-
