# Supplementary material for: Bone metastases and immunotherapy in patients with advanced non-small-cell lung cancer
Source: J Immunother Cancer. 2019 Nov 21;7:316. doi: 10.1186/s40425-019-0793-8 (PMC6868703; doi:10.1186/s40425-019-0793-8)
Supplement: Supplementary file 6 — Additional file 6. PFS and OS in patients treated with nivolumab in second-line in cohort B. [file 40425_2019_793_MOESM6_ESM.doc]

**A.**

**B.**

**Additional file 6: PFS and OS in patients treated with nivolumab in second-line in cohort B**. **A:** PFS was significantly shorter among BoM+ (2.8 months) than among BoM- (4.9 months, p=0.01). **B:** OS was significantly shorter among BoM+ (4.8 months) than among BoM- (8.7 months, p=0.005).
