# Supplementary material for: Bone metastases and immunotherapy in patients with advanced non-small-cell lung cancer
Source: J Immunother Cancer. 2019 Nov 21;7:316. doi: 10.1186/s40425-019-0793-8 (PMC6868703; doi:10.1186/s40425-019-0793-8)
Supplement: Supplementary file 7 — Additional file 7. Efficacy according to clinical characteristics in cohorts B. [file 40425_2019_793_MOESM7_ESM.doc]

**Additional file 7: Efficacy according to clinical characteristics in cohorts B**

| **Category** | **N/Total (%)** | **ORR*** | ***p*** | **mPFS, months** | ***p*** | **mOS, months** | ***p*** |
| --- | --- | --- | --- | --- | --- | --- | --- |
| **ECOG PS 0** |  |  |  |  |  |  |  |
| - Squamous BoM+ | 36/120 (30) | 13.8% | 0.07 | 3.8 (1.4-6.2) | 0.001 | 5.8 (3.4 – 8.2) | < 0.0001 |
| - Squamous BoM- | 98/251 (39) | 28.9% |  | 7.2 (2.2 -12.2) |  | 16.4 (14.2 – 18.6) |  |
| **Liver Metastases** |  |  |  |  |  |  |  |
| - Squamous BoM+ | 25/120 (21) | 4.0% | 0.15 | 2.3 (2.2 – 2.4) | 0.11 | 5.5 (1.8 – 9.2) | 0.48 |
| - Squamous BoM- | 38/251 (15) | 15.8% |  | 2.6 (0 – 5.9) |  | 6.4 (4.1 – 8.7) |  |
| **Brain Metastases** |  |  |  |  |  |  |  |
| - Squamous BoM+ | 16/120 (23) | 12.5% | 0.39 | 2.5 (0.8 – 4.6) | 0.15 | 2.7 (1.5 – 3.9) | 0.03 |
| - Squamous BoM- | 21/251 (8) | 23.8% |  | 6.7 (4.7 – 8.6) |  | 11.7 (1.9 – 21.5) |  |
| **Second line** |  |  |  |  |  |  |  |
| - Squamous BoM+ | 54/120 (44) | 11.1% | 0.09 | 2.8 (2.1-3.5) | 0.02 | 4.8 (3.2-6.4) | 0.003 |
| - Squamous BoM- | 108/251 (43) | 22.2% |  | 4.9 (3.8-6.0) |  | 8.7 (5.5-11.9) |  |

*p value was calculated in CR+PR versus SD+PD
