# Supplementary material for: Bone metastases and immunotherapy in patients with advanced non-small-cell lung cancer
Source: J Immunother Cancer. 2019 Nov 21;7:316. doi: 10.1186/s40425-019-0793-8 (PMC6868703; doi:10.1186/s40425-019-0793-8)
Supplement: Supplementary file 8 — Additional file 8. Univariate and multivariate analyses for PFS in cohorts A and B combined. [file 40425_2019_793_MOESM8_ESM.doc]

**Additional file 9: Univariate and multivariate analyses for PFS in cohorts A and B combined**

|  | **Non-squamous cohort** | | | | **Squamous cohort** | | | |
| --- | --- | --- | --- | --- | --- | --- | --- | --- |
| **Factors** | **Univariate analysis HR (95% c.i.)** | **p** | **Multivariate analysis**  **HR (95% c.i.)** | **p** | **Univariate analysis**  **HR (95% c.i.)** | **p** | **Multivariate analysis**  **HR (95% c.i.)** | **p** |
| Age (≥ 65 vs ≤ 65) | 0.90 (0.80-1.01) | 0.06 | - | - | 1.10 (0.85-1.41) | 0.46 | - | - |
| Gender  (male vs female) | 0.92 (0.82-1.03) | 0.14 | - | - | 0.82 (0.61-1.10) | 0.18 | - | - |
| ECOG PS  1 vs 0  2 vs 0 | 1.37 (1.22-1.54)  2.24 (1.80-2.78) | < 0.0001  < 0.0001 | 1.26 (1.12-1.43)  2.05 (1.63-2.58) | <0.0001  <0.0001 | 1.56 (1.20-2.03)  1.97 (1.19-3.24) | 0.001  0.008 | 1.52 (1.18-1.96)  1.81 (1.12-2.95) | 0.001  0.02 |
| Smoking habits  Current/former vs never | 0.78 (0.68-0.89) | <0.0001 | 0.81 (0.71-0.93) | 0.003 | 0.99 (0.64-1.52) | 0.96 | - | - |
| Brain mets  (yes vs no) | 1.14 (1.01-1.29) | 0.04 | - | - | 0.89 (0.60-1.30) | 0.55 | - | - |
| Liver mets  (yes vs no) | 1.60 (1.40-1.83) | <0.0001 | 1.42 (1.23-1.63) | <0.0001 | 1.37 (1.03-1.84) | 0.03 | - | - |
| Bone mets  (yes vs no) | 1.47 (1.31-1.64) | <0.0001 | 1.37 (1.21-1.54) | <0.0001 | 1.65 (1.30-2.10) | <0.0001 | 1.57 (1.23-2.00) | <0.0001 |
| Previous CT Lines  2 vs 1  >2 vs 1 | 1.01 (0.88-1.16)  1.05 (0.92-1.19) | 0.85  0.49 | - | - | 0.97 (0.74-1.27)  0.89 (0.67-1.20) | 0.82  0.45 | - | - |
