# Supplementary material for: Bone metastases and immunotherapy in patients with advanced non-small-cell lung cancer
Source: J Immunother Cancer. 2019 Nov 21;7:316. doi: 10.1186/s40425-019-0793-8 (PMC6868703; doi:10.1186/s40425-019-0793-8)
Supplement: Supplementary file 9 — Additional file 9. Univariate and multivariate analyses for ORR in cohorts A and B combined. [file 40425_2019_793_MOESM9_ESM.doc]

**Additional file 10: Univariate and multivariate analyses for ORR in cohorts A and B combined**

|  | **Non-squamous cohort** | | | | **Squamous cohort** | | | |
| --- | --- | --- | --- | --- | --- | --- | --- | --- |
| **Factors** | **Univariate analysis OR (95% c.i.)** | **p** | **Multivariate analysis**  **OR (95% c.i.)** | **p** | **Univariate analysis**  **OR (95% c.i.)** | **p** | **Multivariate analysis**  **OR (95% c.i.)** | **p** |
| Age (≥ 65 vs ≤ 65) | 1.29 (0.99-1.67) | 0.06 | - | - | 1.05 (0.60-1.83) | 0.86 | - | - |
| Gender  (male vs female) | 1.16 (0.89-1.53) | 0.27 | - | - | 0.86 (0.45-1.62) | 0.63 | - | - |
| ECOG PS  1 vs 0  2 vs 0 | 0.65 (0.50-0.84)  0.43 (0.23-0.80) | 0.001  0.008 | 0.73 (0.55-0.97)  0.48 (0.25-0.94) | 0.03  0.03 | 0.61 (0.36-1.03)  n.e. | 0.06  n.e. | - | - |
| Smoking habits  Current/former vs never | 2.71 (1.79-4.10) | <0.0001 | 2.74 (1.78-4.15) | <0.0001 | 0.73 (0.30-1.78) | 0.49 | - |  |
| Brain mets  (yes vs no) | 0.86 (0.64-1.16) | 0.33 | - | - | 1.02 (0.43-2.44) | 0.96 | - | - |
| Liver mets  (yes vs no) | 0.47 (0.32-0.69) | <0.0001 | 0.60 (0.40-0.89) | 0.01 | 0.50 (0.21-1.14) | 0.10 | - | - |
| Bone mets  (yes vs no) | 0.45 (0.34-0.60) | <0.0001 | 0.48 (0.36-0.66) | <0.0001 | 0.52 (0.28-0.97) | 0.04 | 0.52 (0.28-0.97) | 0.04 |
| Previous CT Lines  2 vs 1  >2 vs 1 | 1.07 (0.79-1.44)  0.76 (0.55-1.03) | 0.68  0.08 | - | - | 1.02 (0.56-1.86)  0.94 (0.48-1.83) | 0.96  0.85 | - | - |
