# Supplementary material for: Bone metastases and immunotherapy in patients with advanced non-small-cell lung cancer
Source: J Immunother Cancer. 2019 Nov 21;7:316. doi: 10.1186/s40425-019-0793-8 (PMC6868703; doi:10.1186/s40425-019-0793-8)
Supplement: Supplementary file 10 — Additional file 10. Outcome to nivolumab in BOM+ patients according to prior RT. [file 40425_2019_793_MOESM10_ESM.doc]

**Additional file 10. Outcome to nivolumab in BOM+ patients according to prior RT**

|  | **Median OS** | ***p value*** | **Median PFS** | ***p value*** | **ORR** | ***p value*** |
| --- | --- | --- | --- | --- | --- | --- |
| **BoM+/RT+, (N=302)** | **7.1 (95% CI, 5.7-8.5)** | **0.68** | **3.0 (95% CI ,2.8-3.1)** | **0.58** | **10.7%** | **0.26** |
| **BoM+/RT-**  **(N=444)** | **6.6 (95% CI, 5.0-8.2)** |  | **3.0 (95% CI ,2.7-3.3)** |  | **13.4%** |  |
