# Supplementary material for: Bone metastases and immunotherapy in patients with advanced non-small-cell lung cancer
Source: J Immunother Cancer. 2019 Nov 21;7:316. doi: 10.1186/s40425-019-0793-8 (PMC6868703; doi:10.1186/s40425-019-0793-8)
Supplement: Supplementary file 11 — Additional file 11. Early death and early progression in the whole study population according to bone metastases (BoM) and to prior palliative radiotherapy (RT) and in the METROS cohort. [file 40425_2019_793_MOESM11_ESM.doc]

**Additional file 11. Early death and early progression in the whole study population according to bone metastases (BoM) and to prior palliative radiotherapy (RT) and in the METROS cohort.**

|  | **Early Death**  **N/%** | ***p value*** | **Early Progression**  **N/%** | ***p value*** |
| --- | --- | --- | --- | --- |
| **EAP Nivolumab** | | | | |
| **BoM+, (N=746)** | **196 (26.3)** | **<0.0001** | **435 (58.3)** | **<0.0001** |
| **BoM -, (N=1213)** | **203 (16.7)** |  | **518 (42.7)** |  |
| **EAP nivolumab, according to RT** | | | |  |
| **BoM+/RT+, (N=302)** | **76 (25.5)** | **0.69** | **178 (59.7)** | **0.85** |
| **BoM+/RT-, (N=444)** | **120 (26.8)** |  | **260 (58.0)** |  |
| **METROS** | | | | |
| **Overall population, (N=64)** | **3 (4.7)** | **NE** | **6 (9.4)** | **NE** |
| **BoM+, (N=22)** | **1 (4.5%)** |  | **4 (18.2%)** |  |

**NE, not estimable due to the small sample size**
