# Supplementary material for: Bone metastases and immunotherapy in patients with advanced non-small-cell lung cancer
Source: J Immunother Cancer. 2019 Nov 21;7:316. doi: 10.1186/s40425-019-0793-8 (PMC6868703; doi:10.1186/s40425-019-0793-8)
Supplement: Supplementary file 12 — Additional file 12. Treatment related adverse events in cohorts A and B. [file 40425_2019_793_MOESM12_ESM.doc]

**Additional file 11: Treatment related adverse events in cohorts A and B**

|  | **Cohort A** | | | | | **Cohort B** | | | |
| --- | --- | --- | --- | --- | --- | --- | --- | --- | --- |
| **Treatment-related AEs** | **Bone metastases** | | | **No bone metastases** | | **Bone metastases** | | **No bone metastases** | |
|  | | **Any grade**  **n (%)** | **Grade 3-4**  **n (%)** | **Any grade n (%)** | **Grade 3-4 n (%)** | **Any grade n (%)** | **Grade 3-4 n (%)** | **Any grade n (%)** | **Grade 3-4 n (%)** |
| **Any treatment-related AEs** | | **197 (31)** | **44 (7)** | **326 (34)** | **58 (6)** | **31 (26)** | **7 (6)** | **78 (31)** | **14 (6)** |
| **General**  Fatigue/asthenia  Pyrexia  Anorexia | | **62 (10)**  **20 (3)**  **29 (5)** | **11 (2)**  **1 (<1)**  **1 (<1)** | **113 (12)**  **38 (4)**  **27 (3)** | **15 (2)**  **1 (<1)**  **1 (<1)** | **6 (5)**  **4 (3)**  **2 (2)** | **0**  **0**  **0** | **18 (7)**  **6 (2)**  **7 (3)** | **2 (1)**  **0**  **0** |
| **Skin and mucosal**  Rash | | **29 (5)**  **9 (1)** | **3 (1)**  **1 (<1)** | **91 (9)**  **43 (4)** | **7 (1)**  **5 (<1)** | **11 (9)**  **10 (8)** | **1 (1)**  **1 (1)** | **31 (12)**  **21 (8)** | **4 (2)**  **2 (1)** |
| **Gastrointestinal**  Diarrhoea  Nausea/vomiting | | **65 (10)**  **19 (3)**  **26 (4)** | **4 (1)**  **2 (<1)**  **0** | **128 (13)**  **54 (6)**  **43 (4)** | **10 (1)**  **4 (<1)**  **2 (<1)** | **12 (10)**  **7 (6)**  **5 (4)** | **3 (3)**  **3 (3)**  **0** | **15 (6)**  **11 (4)**  **3 (1)** | **1 (<1)**  **1 (<1)**  **0** |
| **Hematologic**  Anemia | | **26 (4)**  **16 (3)** | **7 (1)**  **6 (1)** | **17 (2)**  **6 (1)** | **2 (<1)**  **0** | **2 (2)**  **2 (2)** | **1 (1)**  **1 (1)** | **8 (3)**  **7 (3)** | **0**  **0** |
| **Pain** | | **19 (3)** | **2 (<1)** | **64 (7)** | **6 (1)** | **9 (7)** | **3 (3)** | **10 (4)** | **0** |
| **Hepatic/pancreatic**  Increased transaminase  Increased lipase/amylase | | **30 (5)**  **8 (1)**  **5 (1)** | **11 (2)**  **5 (1)**  **3 (1)** | **38 (4)**  **12 (1)**  **12 (1)** | **14 (1)**  **4 (<1)**  **3 (<1)** | **1 (1)**  **0**  **1 (1)** | **0**  **0**  **0** | **7 (3)**  **6 (2)**  **1 (<1)** | **4 (2)**  **4 (2)**  **0** |
| **Endocrine**  Hypothyroidism  Hyperthyroidism | | **30 (5)**  **13 (2)**  **17 (3)** | **2 (<1)**  **0**  **2 (<1)** | **45 (5)**  **24 (2)**  **20 (2)** | **3 (<1)**  **2 (<1)**  **0** | **3 (3)**  **2 (2)**  **0** | **0**  **0**  **0** | **13 (5)**  **8 (3)**  **5 (2)** | **1 (<1)**  **0**  **1 (<1)** |
| **Respiratory/pulmonary**  Dyspnea  Pneumonitis | | **70 (11)**  **26 (4)**  **14 (2)** | **11 (2)**  **6 (1)**  **4 (1)** | **124 (13)**  **53 (6)**  **17 (2)** | **17 (2)**  **13 (1)**  **5 (<1)** | **1 (1)**  **0**  **0** | **0**  **0**  **0** | **11 (4)**  **7 (3)**  **3 (1)** | **4 (2)**  **2 (1)**  **1 (<1)** |
